# Prevalence of *Cryptosporidium* spp. and *Giardia intestinalis* in Swimming Pools, Atlanta, Georgia

**DOI:** 10.3201/eid1406.071495

**Published:** 2008-06

**Authors:** Joan M. Shields, Elizabeth R. Gleim, Michael J. Beach

**Affiliations:** * Centers for Disease Control and Prevention, Atlanta, Georgia, USA; † CDC Foundation, Atlanta, Georgia, USA

**Keywords:** Cryptosporidium, Giardia, recreational water illnesses, swimming pool, swimming pool contamination, disinfection, hygiene, dispatch

## Abstract

*Cryptosporidium* spp. and *Giardia intestinalis* have been found in swimming pool filter backwash during outbreaks. To determine baseline prevalence, we sampled pools not associated with outbreaks and found that of 160 sampled pools, 13 (8.1%) were positive for 1 or both parasites; 10 (6.2%) for *Giardia* sp., 2 (1.2%) for *Cryptosporidium* spp., and 1 (0.6%) for both.

*Giardia* sp. and *Cryptosporidium* spp. are gastrointestinal parasites spread through the fecal-oral route. In 2003–2004, these parasites were responsible for 61.2% (*Cryptosporidium* spp. 55.6%; *Giardia* sp. 5.6%) of gastroenteritis outbreaks associated with treated swimming venues (e.g., swimming pools, water parks) in the United States (*1*). *Cryptosporidium*’s key role in these outbreaks is likely because of its small size, low infectious dose (*2*), and high tolerance to chlorine (*3*), which is the major disinfectant used in swimming pools. Despite frequent outbreaks, little is known about these parasites’ occurrence in swimming pools in the absence of outbreaks. Although the frequency of contamination is unknown, 4.4% of formed feces recovered from non–outbreak-related pools were positive for *Giardia* sp. and 0 were positive for *Cryptosporidium* spp. (*4*). In the Netherlands, 7 pools sampled for >1 year had a prevalence of 5.9% for *Giardia* sp., 4.6% for *Cryptosporidium* spp., and 1.3% for both pathogens (*5*). In Italy, 1 study found 28.6% (2/7) of tested pools were positive for both *Giardia* sp. and *Cryptosporidium* spp. (*6*) and another study found 40% (4/10) of tested pools positive for either parasite (*7*). No data exist on the occurrence of these parasites in US pools. Further data on pool contamination would reinforce existing US pool codes and support code changes designed to reduce the level of parasite contamination, particularly chlorine-resistant *Cryptosporidium* spp.

During the past 2 decades, *Cryptosporidium* spp. and *Giardia* sp. have been associated with increasing outbreaks of swimming-associated gastrointestinal illness in the United States; *Cryptosporidium* spp*.* is emerging as the leading cause of swimming pool-associated outbreaks of gastrointestinal illness (*1*). However, the baseline prevalence of contamination in non–outbreak-associated swimming pools is incomplete.

## The Study

A convenience sample of 160 public swimming pools from 2 metropolitan Atlanta, Georgia, counties was used to collect filter backwash samples for parasite examination during a 7-week period (late August–October 2006). Information on age of swimmers, pool type, pool size, and number of swimmers was gathered. No facility identifiers were assigned.

Filter backwashing is a cleaning process by which the water flow through the filter is reversed so that accumulated debris trapped in the filter is dislodged and directed to waste. Filter backwash therefore tends to contain more concentrated pathogens than does pool water. All selected pools had a sand filter (most public pools in the metropolitan Atlanta area use sand filters) and had been used by swimmers before the backwash cycle and sample collection began.

One-liter samples of filter backwash were collected in wide-mouthed plastic bottles shortly after the filter flow grew turbid and were transported and stored at 4°C before flocculation. The samples were calcium carbonate flocculated within 2 weeks, typically within a few days, after collection (*8*). Pellets were stored in DNase, RNase-free, sterile microcentrifuge tubes. DNA was extracted from 250–350 mg of each pellet by using a FastPrep DNA kit (MP Biomedical, Solon, OH, USA); 20 μL of polyvinyl pyrolidone (FW 40,000; Fisher Scientific, Pittsburgh, PA, USA) was added to the CLS-VF buffer provided in the kit. Final purification used a QIAquick spin column kit (QIAGEN, Valencia, CA, USA).

Real-time qPCR used the Stratagene Mx3000P thermocycler (Stratagene, La Jolla, CA, USA) and the triplex PCR reaction and amplification protocol described for *Entamoeba histolytica*, *Giardia intestinalis*, and *Cryptosporidium* spp. (primers and probe amplify both *C. parvum* and *C. hominis*) with a reported sensitivity and specificity of 100% (*9*). DNA from *E. histolytica* was added to each sample as a positive internal control. Sample inhibition was alleviated by repeating the qPCR with 4.7 μg/μL of bovine serum albumin (Sigma, St. Louis, MO, USA).

Of the 160 filter backwash samples collected, 13 (8.1%) were positive for 1 or both parasites; 10 (6.2%) were positive for *G. intestinalis*; 2 (1.2%) were positive for *Cryptosporidium* spp.; and 1 (0.6%) was positive for both pathogen genera. Because of the small amount of target DNA, speciation was not possible with most samples. However, 1 *C. hominis* positive sample was identified.

The Table summarizes parasite prevalence by age of swimmers, pool type, pool size, and number of swimmers. Although 117 (73.1%) of all pools tested were commonly used by children (28 were designated for children only, 89 for children and adults), these pools accounted for 12 (92.3%) of 13 positive pools sampled. In comparison, 43 (26.9%) of 160 pools designated for adult use were associated with 1 (7.7%) of 13 positive pools. Of the positive samples, 10 (76.9%) of 13 were found in community pools, although community pools accounted for 40% (64/160) of pools assayed. Small-volume pools (<50,000 gallons) comprised 72 (45%) of 160 sampled pools but accounted for 9 (69.2%) of 13 positive samples. Similarly, pools with <75 swimmers per week comprised 42.5% of pools but accounted for 10 (76.9%) of 13 positive samples.

**Table:** Pathogen distribution in 13 *Cryptosporidium*- and  *Giardia*-positive swimming pools (n = 13)

Characteristic	% (n/N)	Type of parasite*
Age of swimmers†		
Children	10.7 (3/28)	2 G, CG
Adults	2.3 (1/43)	G
Mixed	10.1 (9/89)	7 G, C, Ch
Total	8.1 (13/160)	
Pool type‡		
Community	15.6 (10/64)	7 G, CG, C, Ch
School	4.8 (1/20)	G
Health club	0 (0/25)	
Apartment	3.9 (2/51)	2 G
Total	8.1 (13/160)	
Pool size (x1,000 gallons)		
<5	22.2 (4/18)	3 G, CG
6–50	9.3 (5/54)	4 G, Ch
51–100	0 (0/40)	
101–200	11.4 (4/35)	3 G, C
>200	0 (0/13)	
Total	8.1 (13/160)	
No. swimmers/bathers per week		
1–75	14.7% (10/68)	7 G, CG, C, Ch
76–200	7.4% (2/27)	2 G
201–500	2.9% (1/35)	G
>500	0% (0/30)	
Total	8.1% (13/160)	

*G, *Giardia intestinalis; *C, *Cryptosporidium* spp.; Ch, *C. hominis;* CG, both *G. intestinalis* and *Cryptosporidium* spp. †Children defined as persons <16 y of age; adults defined as persons >16 y of age. ‡Residential and hotel/motel swimming pools were excluded from the study.

Although this study was small and the power low, estimates of the prevalence odds ratio (POR) were calculated. Those associations that were statistically significant for finding protozoa-positive samples were as follows: community pools (POR 5.7, 95% confidence interval [CI] 1.5–21.8) and weekly number of swimmers of <75 (POR 5.1, 95% CI 1.4–19.4). Although the positive parasite sample prevalence was higher in pools frequented by children and adults (10.1%) than in pools designated for adults only (2.3%), the POR was not significant (POR 4.8, 95% CI 0.6–38.1), likely because of the small sample size.

Prior analysis of 22,131 US pool inspections demonstrated that children’s pools had an increased incidence of critical pool code violations perhaps because their smaller volumes and depths make maintaining appropriate levels of disinfectant more difficult (*4*,*10*). In addition, younger children may be more likely to contaminate recreational water as a result of being incontinent or having higher levels of perianal fecal contamination (*11*). This finding necessitates greater vigilance in maintaining water quality for this population because they are more likely to contaminate the water and are more vulnerable to the severe effects of diarrheal illnesses.

## Conclusions

This study is a snapshot of contamination at the end of the swim season. Although an earlier sampling schedule may have detected more contamination, these findings suggest that contamination events in some pool types or with some swimmer compositions may be relatively common during the swim season. The prevalence of contamination found by this study is difficult to compare with that found by other studies that focus on serial samples from a small number of pools. However, the key finding, parasite detection, is repeated in all the studies cited (*5*–*7*). The risk for disease transmission is difficult to ascertain because most studies, including this one, have not measured viability of the parasites recovered from water or filter backwash. However, intact *Cryptosporidium* oocysts observed following hyperchlorination to inactivate the parasite are commonly noninfectious (M.J. Arrowood, pers. comm.).

This study is limited by having a small sample size, by being a convenience sample, and by using backwash collected from pools with a single filter medium (i.e., sand) exclusively. In addition, the sensitivity and specificity of PCR detection in pool-associated backwash samples is unknown, although positive and negative controls reacted appropriately. Although these deficiencies would likely lead to underestimates of the prevalence of parasites in this sample, clearly such study results are neither generalizable to all types of pools nor an accurate measure of national contamination levels. However, despite these deficiencies, the finding of swimming pool filter contamination by *Giardia* sp. and *Cryptosporidium* spp*.* is key and reinforces the need for continued emphasis on improving pool operation and maintenance (e.g., preventive hyperchlorination or flocculation on a routine basis). These improvements should also include consideration of supplementary inline disinfection systems known to inactivate *Cryptosporidium* spp. (e.g., ultraviolet light, ozone) and other pathogens (*3*,*12*–*14*). These data also underscore the need for the general public, particularly immunocompromised persons, to understand recreational water–associated illness transmission and adopt healthy swimming habits (e.g., no swimming when ill with diarrhea, no swallowing of pool water, improved hygiene [*15*]) that are needed to reduce the risk for pathogen transmission.
